# Is It Legitimate for Society to Intervene in the Way Citizens Live Their Lives When the Cost of Health Care Has to Be Borne by the General Public?—General Considerations and Special Implications During the Covid-19 Pandemic

**DOI:** 10.3389/fpubh.2021.653923

**Published:** 2021-09-22

**Authors:** Christoph Edlinger, Dominic Klein, Michael Lichtenauer

**Affiliations:** ^1^Department of Cardiology, Heart Center Brandenburg, Bernau/Berlin, Germany; ^2^Brandenburg Medical School (MHB) “Theodor Fontane”, Neuruppin, Germany; ^3^Division of Cardiology, Department of Internal Medicine 5, Hospital Favoriten, Vienna, Austria; ^4^Division of Cardiology, Department of Internal Medicine II, Paracelsus Medical University, Salzburg, Austria

**Keywords:** autonomy, restrictions, COVID-19, sick person's role, public health

## Abstract

Over the last few decades, the perception of disease has changed significantly. In the concept of the sick person's role it should be the aim of every person to keep health at a good level for as long as possible. Several examples can be found where, however, a disease can be caused or worsened by a person. Examples include unhealthy diet, alcohol consumption leading to atherosclerosis and diabetes, or smoking, leading to lung cancer and COPD. There are also other appropriate examples where there is a potential for conflict between the autonomy of the individual and health. Improving public health should be the main objective of any health system. However, the more the impact is on personal freedom (and there is no extraneous danger), the more an attempt should be made to achieve this through the motivation of each individual to support the desire for a healthy lifestyle, rather than through legal prohibitions or penalties. The situation is even more complex in the case of the Covid-19 pandemic. In this context too, personal freedom is restricted in many areas and some people feel, for example, that compulsory masks or the prohibition of large crowds are serious encroachment on their autonomy. However, even in this case, the risk of possible external threats from the spread of the virus outweighs the right to personal choice and freedom. To sum up, it is necessary to balance the two principles - autonomy and interference in them in the interests of public health.

## Introduction

The health system of several European countries is based on a social security system and is financed by compulsory insurance for all citizens. Such financing models can be found throughout Europe (e.g., very similar systems exist in Germany, France, Austria and the Benelux countries) and date back to the introduction of compulsory health insurance in 1883 by Otto von Bismarck (1). In contrast, there are still models of a national health service financed by taxes, such as in Great Britain, Italy, Ireland, Denmark and Portugal, and predominantly privately financed models, such as in the USA. In an international comparison of these models, the Austrian health care system for example is regarded as rather expensive, but it is also among the best in terms of the quality of health care services. In this model of health financing, all citizens (or employers) pay a percentage of their income (or pension) into the health system or into the statutory health insurance funds in the form of contributions. In return, the costs of treating illnesses are covered by the system (2). There are also deductibles in some areas, such as the prescription fee for medication or dental treatment. The aim of this system is to create a social balance and to ensure that all sections of the population receive the same high quality health care. The aim of such a system is to compensate for social differences, for example in income, social environment, education and origin, all determinants of long-term health, and to be able to offer fair health care to the entire population. In addition, there is genetics, which means that the risk of a disease is unequally distributed in the population. Even though this system attempts to provide health care as broadly as possible for all strata of the population with low barriers, there are still potential conflict zones and moral hazards (3). The central theme of this essay is to present the conflict question “Since the costs of health care must be borne by the general public, is it legitimate for society to intervene in the lifestyle of the citizen?”

Over the last decades, the picture and the view of diseases have changed. Whereas in the past, for example in the Middle Ages, a disease was seen as God's punishment, the concept of disease changed in the following centuries and a disease was often seen later as a fateful process. The sick person was therefore not responsible for his/her condition in the eyes of society. The American sociologist Talcott Parsons explored this concept or viewpoint and described it in his treatise on the sick role (4). According to this concept, the sick role goes hand in hand with rights but also duties. These rights include the right to be removed from the normal social role (for example, the right to sick leave), the right to be accepted (in the sense of medical treatment), and the right not to be responsible or liable for his/her present condition. On the other hand, the sick person is also attributed duties, namely that the sick person should seek to recover and seek medical assistance. However, this model of the sick person's role is not ideally applicable in all areas and has led to criticism. This model is more tailored to acute illnesses and less appropriate in the setting of chronic illnesses or disabilities. Furthermore, the role of the sick person(s), which is seen as rather passive, and the view that the individual should not be responsible for the illness, also attracted criticism. Thus, several examples can be found in which an illness can very well be influenced or triggered by the individual. Examples include unhealthy diet and the occurrence of atherosclerosis or diabetes, smoking and most forms of lung cancer or COPD. Even as a counter-example, a study by Chalfont and Kurtz in 1971, which looked at alcohol addiction, showed that people with alcohol addiction are seen by society as responsible for their illness and stigmatized, or that alcohol addiction is sometimes not seen as a disease at all (5). Examples like these therefore do not fit into the model postulated by Parsons.

In modern society, the concept of illness has continued to change and the influence of the individual on health and illness is becoming increasingly important. Based on the results of medical research in recent decades, the connection between health and illness has been deciphered in detail in many areas. A suitable example of this would be atherosclerosis research, where studies have proven the various influencing factors such as nutrition, inflammation and family history (6). As mentioned above, one of the key driving factors is diet, as it has been shown that high blood lipid levels can lead to a rapid progression of the disease, which can significantly increase the risk of heart attack and stroke. Although decades of research and frequent media coverage of the most common risks, such as poor nutrition and lack of exercise, have led to the conclusion that every person should be aware of the lifestyle that leads to these diseases, a substantial part of the population refuses to accept these insights. This is partly due to traditional lifestyles and habits and certainly also to a certain amount of neglect of the risks of disease.

## Smoking and Lung Disease

Another appropriate example is smoking. Especially in Austrian politics, a general ban on smoking in restaurants and bars with its introduction and abolition has been a frequent topic of dispute in recent years. In the case of smoking, too, the health risks have long been known and a clear association with the occurrence of lung carcinomas and COPD, but also cardiovascular diseases, has been demonstrated. Despite this knowledge, many people are exposed to this risk every day through the consumption of cigarettes. Moreover, passive smoking can also affect uninvolved people.

At the same time, there is an inconsistent political line on the smoking ban in restaurants and bars within Europe.

For example, Berlin's gastronomic establishments are allowed to position themselves either as “smoking establishments” or as non-smoking establishments. The idea is that the customer decides for him/herself and his/her health which type of establishment he/she wants to visit.

Other countries such as Italy or Ireland, where smoking has been banned in public places for many years now, had a much stricter approach. Italy in particular, played a pioneering role in the inner-European comparison in terms of non-smoker protection. Examples include information campaigns, banning advertising for cigarettes or the placing of large-format warnings on tobacco products.

Nevertheless, public measures have been taken in many countries in recent years to reduce the risks of tobacco consumption.

## Alcohol Consumption

Alcohol consumption offers a similar example. In this case too, it is known that high alcohol consumption can lead to addiction and mental and physical illness (for example, cirrhosis of the liver or heart failure). In society, alcohol consumption has been a social convention for centuries and is an accepted consumer good in all walks of life. This ranges from excessive alcohol consumption by young people as a form of initiation rites (keyword binge drinking) to wine tastings at all ages and social settings. As already mentioned above, alcohol addiction continues to be a repressed and stigmatized disease, as described by Chalfont and Kurtz in the 1970s. This may be due to the fact that alcohol occupies a central place in Western society, even more so than smoking, and is ubiquitous as a consumer product in shops and restaurants.

In this context, there is a clear discrepancy within Europe. Central European countries such as Austria, Germany or the Czech Republic have always been at the forefront of per capita consumption, whereas consumption in southern European regions such as southern Italy or Spain is comparatively moderate.

In the Scandinavian countries and in Finland, there have been clear restrictions for many years, insofar as high-percentage alcoholic beverages are only sold in a few and are moreover taxed at a very high rate. On the one hand, this offers the advantage of government control, especially when it comes to the consumption behavior of minors. However, we know from times of prohibition in the USA that the danger of black market trade or even illegal own production might increase here, so that in many countries there is an increasing focus on targeted prevention.

Even though there have been repeated efforts in recent decades to launch educational campaigns to inform people about the dangers of alcohol, there have been few socio-political measures aimed at reducing alcohol consumption, with the exception of the blood alcohol limit in road traffic. In contrast to tobacco products, there are also hardly any restrictions on the sale or advertising of alcohol products, possibly because they occupy such a central social and economic place in many western countries.

## Special Implications During the Covid-19 Pandemic

The subject of this essay is whether society can intervene in the way people live their lives when it comes to the health of the general public, which, after all, has to bear the bulk of the costs due to compulsory insurance or government-funded healthcare. In this context, however, it is not only a question of the costs of illness, but also of whether, and if so to what extent, health policy makers should and can intervene in the lives of citizens when it comes to maintaining or improving public health. The Covid-19 pandemic is a very recent example in this context. No other health crisis in recent decades, or even centuries, has had such a profound impact on the daily life of the world's population. Governments all over the world tried to stop the spread of the virus and the associated risk of infecting large parts of the population by means of a lock-down of social life (closing of shops, cancellation of public events) and instructions on how to behave in public (social distancing and compulsory masks in shops and public transport). A year ago, it would hardly have been conceivable that our daily life could have changed so fundamentally and that such far-reaching measures on the part of the government would have been necessary to put into practice. Although the Covid 19 crisis is not yet over, it can be assumed that these farreaching measures, which also strongly affect the autonomy of the individual(s), could prevent a faster spread of the Sars CoV-2 virus and a resulting overloading of the health care system in the affected countries. In our opnion, the actions of those in power in this current pandemic crisis represent a particularly remarkable example of how far-reaching and stringent measures, which of course also have a negative impact on the autonomy of the individual, are being used to preserve the well-being and health of the general public. As is probably the case in most situations where such cuts in personal freedom occur, resistance to government measures (corona parties, demonstrations and conspiracy theories in social networks) has formed in some sections of the population. Such measures therefore always represent a tightrope walk between encroachments on personal freedom and desired positive effects on the health of the individual or individuals and the general public.

However, it is not only the COVID pandemic or the acute infection itself that raises a multitude of ethical questions. In many countries, opinions around COVID-19 vaccination are currently dividing societies. In particular, many nations are currently discussing compulsory COVID-19 vaccination. The challenging question here is - can the state impose compulsory vaccination on its citizens in order to achieve herd immunity? In this context, it must be considered that a vaccination of young adults or even children is also necessary, whose risk course for a severe course of the disease is considered to be comparatively low.

In this context, therefore, a balancing of interests takes place. There is a conflict between the “good of health (the general public)” and the “good of autonomy”. In philosophy and ethics, a “good” represents a desired goal of human endeavor. The philosopher Plato described three different forms of goods, namely intrinsic goods in the sense of pleasure experiences, which are primarily striven for because of themselves and not because of their consequences, and extrinsic goods such as medical therapy, which are striven for because of their consequences and not for their own sake. In addition, there are goods that have both intrinsic and extrinsic values, such as health. Health is desired both for its own sake and for the sake of its consequences, as it provides momentary well-being and is the prerequisite for pursuing our goals in the future. Autonomy is also such an intrinsic and extrinsic good, since its presence is important both for our present well-being and for the realization of our future desires. It is precisely here that there is a particular potential for conflict in the context of general health vs. autonomy.

Nevertheless, the preservation of personal decision-making ability and autonomy is of great importance in medicine. If a patient is undergoing medical treatment due to a disease, the doctor treating him/her will prescribe a therapy or operation in order to achieve a cure or at least an improvement of the current condition. However, the decision whether the patient agrees to this recommendation is solely his/her responsibility. The doctor can only inform the patient about the consequences of the illness, the course of the therapy and possible risks of action or inaction but cannot force the patient to undergo therapy or surgery. If the patient refuses treatment, he/she can also confirm this in writing by submitting a so-called reverse voucher. This means that the patient renounces treatment on his/her own responsibility and represents a personal decision that must be taken into account by the doctor. On the other hand, doctors cannot be forced to carry out a medical intervention if the patient wants or demands it, but there is no medical indication for it. One example would be plastic/aesthetic, non-reconstructive surgery. Another area of tension is abortion. Here too, the doctor is not obliged to carry out an abortion if it is against his/her ethical or religious understanding. There is, therefore, also a right of autonomy on the part of the doctors. These differences also represent the central issues in medical ethics. The medical ethicists Beauchamp and Childress established four basic principles of medical practice in their research at Georgetown University (7). These four principles of ethical action in medicine include the patient's right to self-determination (right to autonomy), the principle of avoiding harm, the well-being of patients and the goal of social justice.

In this context there are also regional differences which are based on different philosophical attitudes. While in the European countries the attitude of mind is based on Immanual Kant's philosophy and assumes autonomy equally distributed on both sides, in the USA the right of the patient to choose is more widespread. This difference probably developed not only because of other philosophical models but also because of other health care structures, as health in the USA is more a matter of personal choice due to the predominance of private insurance models than in Europe with compulsory health insurance. Therefore, in the USA, the good “health” is more determined by financial factors of the individual, while on the other hand, in some areas the sick person has more freedom of choice regarding medical therapy (right of choice), as long as he or she can afford it or is insured for it.

As mentioned above, the attitude of mind in medicine in Europe goes back more to the philosophical views of Kant. Kant derived the concept of human dignity primarily from the autonomy of the human being. The individual has a choice, he/she can decide how he/she wants to act, and his/her decision depends on his/her moral and ethical values. Kant formulated the categorical imperative as the fundamental principle of ethical action. This is: “Act only according to that maxim whereby you can, at the same time, will that it should become a universal law” (8). According to Kant, personal freedom is of paramount importance, but only to the extent that it does not violate or restrict the freedom or rights of one or another. Consequently, the latter must have a social compatibility of his/her own actions. The principle of the golden rule, which is often confused with Kant's categorical imperative, goes back even further, but in linguistic usage it is expressed with the phrases “Treat others as you would like to be treated by them” or, conversely, “Do not do to others what you do not want them to do to you” is even more common.

## Discussion

**Figure 1:**
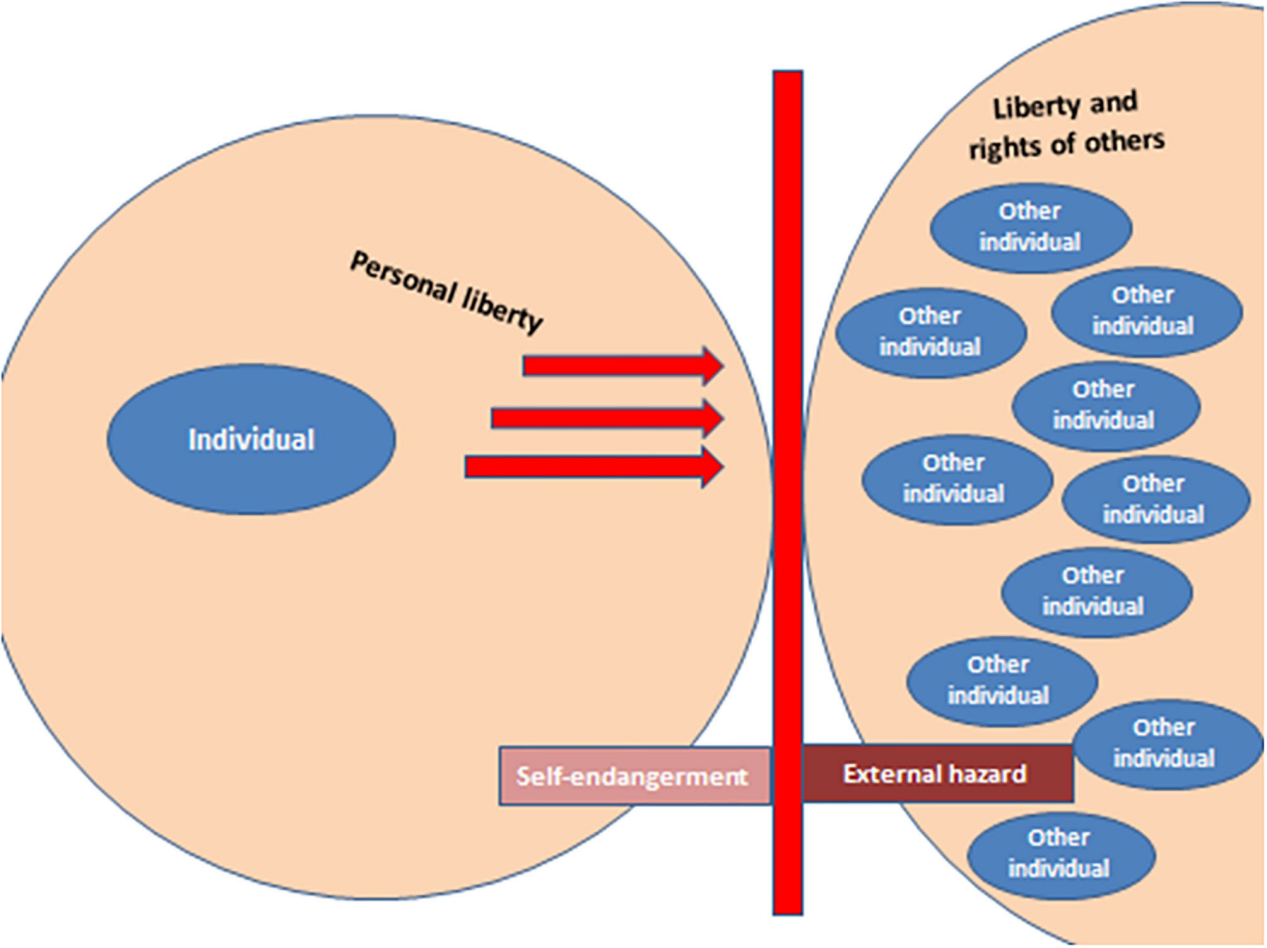
The rights to autonomy and personal freedom generally go so far (including possible self-endangerment) as to reach the area of external danger to others [autonomy up to the external border of the other(s)].

When we look at the points of conflict between autonomy and public health, a red line can be drawn where the autonomy of the individual restricts the freedom or rights of others or the general population (see Figure 1). For example, although individuals are not forbidden to consume alcohol in large quantities that endanger their own health, the safety and physical integrity of other people may be endangered. In road traffic, for example, legal regulations are in place to prevent injuries as far as possible (e.g., blood alcohol limits, driving bans). The situation is similar with smoking. Although smokers are made aware of the risks of tobacco consumption through information on cigarette packets, smoking itself is not prohibited despite the known health risks, and doing or not doing so is the free, personal decision and autonomy of the individual. Here too, however, legal provisions only come into play as soon as a possible danger to others can arise, for example through passive smoking in restaurants. Passive smoking by children in the smoker's own four walls is certainly a gray area in this context, as although others/underage persons can also be endangered here, the right to personal freedom is more important in the private sphere than in public. In these two examples, legislation intervenes on behalf of the general public and its health in the rights to freedom of the individual(s) as soon as a mere self-endangerment can lead to a third-party risk. Another example is nutrition. Even though it is known that, as mentioned above, poor nutrition can lead to the development of diseases, in this case, however, it is primarily self-endangerment that exists. Excessive consumption of processed meat products or fast food in general, cannot cause a foreign hazard (apart from the ban on consumption of these in public transport, even if the foreign hazard in this case is of a more olfactory nature). It can therefore be assumed that in such cases there have not yet been any efforts to intervene in the diet of the population directly for the benefit of health through legal measures.

The situation is different in the case of the Covid-19 pandemic. Here, too, personal freedom is restricted in many areas and some people feel, for example, that compulsory masks or the prohibition of large crowds are a serious encroachment on their autonomy. However, even in this case, the risk of possible external threats from the spread of the virus outweighs the right to personal choice and freedom. To sum up, it is necessary to balance the two principles—autonomy and interference in them in the interests of public health. In a State of solidarity, it is up to each individual to decide how to manage his/her own health, as long as this does not create risks for others. Even if the costs of the health system are borne by the general public, this dilemma must seek to strike a balance between personal freedom or even possible behavior that is not beneficial to health and the best possible health of the population as a whole. The goal of improving public health should be one of the main objectives of any government. However, the more the impact is on personal freedom (and there is no extraneous threat), the more efforts should be made to achieve this through information campaigns aimed at the intrinsic motivation of the individual(s), thus supporting the desire for a healthy lifestyle, rather than through legal prohibitions or penalties.

## Data Availability Statement

The original contributions presented in the study are included in the article/supplementary material, further inquiries can be directed to the corresponding author/s.

## Author Contributions

ML developed the concept and the first draft of the manuscript. CE and DK revised the manuscript. All authors contributed to the article and approved the submitted version.

## Conflict of Interest

The authors declare that the research was conducted in the absence of any commercial or financial relationships that could be construed as a potential conflict of interest.

## Publisher's Note

All claims expressed in this article are solely those of the authors and do not necessarily represent those of their affiliated organizations, or those of the publisher, the editors and the reviewers. Any product that may be evaluated in this article, or claim that may be made by its manufacturer, is not guaranteed or endorsed by the publisher.

## References

[1] Hänlein A Tennstedt F Winter H Ayaß W. Quellensammlung zur geschichte der deutschen sozialpolitik 1867 bis 1914, i. Abteilung: Von der reichsgründungszeit bis zur kaiserlichen sozialbotschaft (1867-1881), 5. Band: Gewerbliche unterstützungskassen, ii. Abteilung: Von der kaiserlichen sozialbotschaft bis zu den februarerlassen wilhelms ii. (1881-1890), 5. Band: Die gesetzliche krankenversicherung und die eingeschriebenen hilfskassen, iii. Abteilung: Ausbau und Differenzierung der Sozialpolitik seit Beginn des neuen kurses (1890-1904), 5. Band, die gesetzliche krankenversicherung. Mainz.

[2] Morduch J. Economics and the Social Meaning of Money. Princeton, NJ: Princeton University Press (2017).

[3] Saez E Stantcheva S. Generalized social marginal welfare weights for optimal tax theory. In: National Bureau of Economic Research, NBER Working Paper Series, Cambridge, MA (2013). 10.3386/w18835

[4] Parsons T. The Social System. London, UK: Free Press (1951).

[5] Chalfont HP Kurtz RA. Alcoholics and the sick role: Assessments by social workers. J Health Soc Behav. (1971) 12:66–71. 10.2307/2948455 5572818

[6] Paoletti R Poli A Cignarella A. The emerging link between nutrition, inflammation and atherosclerosis. Expert Rev Cardiovasc Ther. (2006) 4:385–93. 10.1586/14779072.4.3.385 16716099

[7] Beauchamp TL Childress JF. Principles of Biomedical Ethics. Oxford, UK: Oxford University Press (2008).

[8] Kant I. Groundwork of the Metaphysic of Morals. Cambridge, UK: Cambridge University Press (1785/1993).

